# Is There Potential Clinical Utility in Reporting Variants of Uncertain Significance From Prenatal Sequencing?

**DOI:** 10.1002/pd.70192

**Published:** 2026-06-18

**Authors:** A. Gibbs, R. Braham, V. Ramachandran, R. Roberts, C. Willison, T. Ashraf, J. Cobben, A. Gardham, M. Holder‐Espinasse, T. Homfray, S. G. Mehta, D. Tapon, P. Vasudevan, N. J. Chandler

**Affiliations:** ^1^ NHS North Thames Genomic Laboratory Hub Great Ormond Street Hospital for Children NHS Foundation Trust London UK; ^2^ Department of Clinical Genetics Great Ormond Street Hospital for Children NHS Foundation Trust London UK; ^3^ Centre for Fetal Care Queen Charlotte Hospital ICHT NHS & Imperial College London UK; ^4^ Department of Clinical Genetics Guy's Hospital London UK; ^5^ Department of Medical Genetics St George's University of London London UK; ^6^ Department of Clinical Genetics Addenbrooke's Hospital Cambridge UK; ^7^ Queen Charlotte's & Chelsea Hospital Imperial College Healthcare NHS Trust London UK; ^8^ Department of Clinical Genetics University Hospitals of Leicester Leicester Royal Infirmary Leicester UK; ^9^ Genetics and Genomic Medicine UCL Great Ormond Street Institute of Child Health London UK

**Keywords:** multidisciplinary team, prenatal, sequencing, variants of uncertain clinical significance

## Abstract

**Objective:**

Prenatal sequencing of fetuses with abnormalities detected on imaging is expanding globally. Debate continues over whether variants of uncertain significance (VUS) should be reported prenatally, with some recent national position statements opposing this. In England, VUS that fit the fetal phenotype and require little further evidence for upgrade are discussed at multidisciplinary team (MDT) meetings to determine whether to report. We review the VUS reported by one English laboratory that provides prenatal sequencing to half of England.

**Method:**

The laboratory's database was searched from 01/10/2020 to 30/04/2025 to ascertain all cases where a VUS was reported. Pregnancy outcomes were obtained from local clinical teams to determine whether the VUS status had been resolved.

**Results:**

VUS were reported in 41/881 fetuses sequenced. Follow‐up data were available for 38/41 cases, of which 23 were subsequently upgraded to likely pathogenic and 1 downgraded to likely benign. The most common reason for upgrade was new information from post‐mortem or postnatal review (17/23).

**Conclusion:**

There is clinical utility in reporting VUS from prenatal sequencing following MDT discussion. Although reporting VUS leaves parents with uncertainty, follow up (including post‐mortem when applicable) resolves this in over 50% of cases (79% of cases re‐examined pre‐ or postnatally).

## Key Summary

- What's already known about this topic?
  ◦ Reporting pathogenic and likely pathogenic variants that explain the fetal phenotype has become common practice in prenatal sequencing.
  ◦ Controversy exists regarding the reporting of variants of uncertain significance (VUS) in the prenatal setting.
- What does this study add?
  ◦ We provide evidence that prenatal VUS reported following a Multi‐Disciplinary Team discussion has an over 50% likelihood of being subsequently upgraded to likely pathogenic and should therefore be reported.

## 1 Introduction

Ultrasound scanning in pregnancy from the first trimester onwards can identify fetal structural abnormalities [1] that may indicate an underlying genetic condition. Prenatal sequencing, often in the form of exome sequencing (ES) [2], has become a key component of fetal genetic testing in many countries for fetuses with abnormalities detected on ultrasound that may have a genetic etiology [3]. Variants identified post‐filtering are classified regarding the likelihood of pathogenicity in accordance with laboratory policies that normally utilise either the Association for Clinical Genomic Science (ACGS) [4] or American College of Medical Genetics and Genomics (ACMG) [5] recommendations. Classification incorporates many forms of evidence, including comparing the subject's phenotype to those of individuals known to have the disease in question (which can be limited in a fetus without a fully developed phenotype), computational predictions of the variant effect, frequency of the variant within general population databases (e.g., gnomAD [6]), functional characterisation of the variants and the presence of the variant in affected patients either in the literature or databases (e.g., ClinVar [7]). These guidelines describe a framework for classifying variants as “pathogenic”, “likely pathogenic”, “uncertain significance”, “likely benign” or “benign” based on a Bayesian framework. “Pathogenic” and “likely pathogenic” variants are defined as having at least a 90% likelihood of being disease causing and are reported when recognised. Variants of uncertain significance (VUS) have a likelihood of pathogenicity ranging from 10% to 89%, but “hot” VUS that require only minimal further information to be upgraded to likely pathogenicity are identified in around 10%–30% of prenatal cases undergoing ES [8, 9, 10]. Reporting these is controversial in all settings but particularly prenatally, where reporting could lead to the termination of a potentially unaffected fetus, *or not reporting to the birth of a child with severe disability*. VUS may be seen more frequently in the prenatal setting as phenotypes are not as complete before birth, for example, we cannot assess intellectual development, hearing, sight etc. Furthermore, we know that some phenotypes evolve over gestation [11, 12]. Current ACGS guidelines [4] do not differentiate between prenatal and postnatal cases.

Receiving a VUS report has been described as “very stressful” by both receiving parents [13] and reporting doctors [14] alike. In England, there has been extensive evaluation of the views of both parents undergoing prenatal sequencing and the healthcare professionals delivering the rapid fetal sequencing service [15]. One healthcare professional interviewed felt that VUS reporting when “there's no pointers on ultrasound or in their history to the meaning of that variant … is a bit wishy washy and I find they (the parents) just can't process it. I think the cases I've seen, they've had termination anyway because … this is just too much now.” [14] The International Society for Prenatal Diagnosis (ISPD) has released its position on VUS reporting: “some laboratories may report variants of uncertain significance in strong candidate disease genes for the fetal phenotype, for example, in an autosomal recessive gene which is relevant for the fetal phenotype, when a pathogenic (or likely pathogenic) variant is inherited from one parent along with a variant of uncertain significance from the other parent” [16]. Nevertheless, national positions on VUS reporting vary. Canada has issued guidance supporting reporting of VUS in “rare exceptional circumstances … with very strong evidence of pathogenicity or a VUS in trans with a pathogenic … variant … known to cause a recessive disease.” [17] Conversely, a recent national position statement from France stated categorically that “VUS should not be reported to would‐be parents during the pregnancy” [18]. As well as concern for termination decisions based on prenatal VUS reporting, Cogan et al. [18] feared that they could lead to demand for prenatal diagnoses in future pregnancies. This is something that we have also observed in our clinical practice and indicates that we should make all efforts possible to clarify the status of any VUS reported.

Fu et al. found that performing trio ES (involving the fetus' biological parents in addition to the fetus) reduced the rate of reporting VUS by over 60% [19]. Another benefit of trio sequencing is that it facilitates the identification of compound heterozygosity by allowing phasing of two detected variants [20]. However, trio sequencing can also lead to the identification of secondary or incidental findings in parents, such as hereditary cancer predisposition for example *BRCA2* variants. The rate of identifying these additional findings ranges from 6% to 16% in published literature [10, 19]. Determining how to report these can be problematic and induce anxiety in the parents. The possibility of these findings should be discussed with parents while obtaining consent for ES to enable them to decide whether they would want to know about any incidental finding if identified [21]. Incidental findings in parents are reduced by only analysing those variants found in the fetus in parents [22]. Discussion of these findings is excluded from this paper as their implications and management are different from VUS.

In England, where trio (rather than single/duo) ES is carried out whenever possible, some VUS are reported to parents. Common practice is for VUS that fit the fetal phenotype and require little additional evidence for upgrade to “likely pathogenic” or “pathogenic” (often termed “warm” or “hot” VUS) to be discussed by a multidisciplinary team (MDT) for determination of whether the VUS should be reported to the parents. The MDT consists of clinical geneticists and clinical scientists, *often joined by fetal medicine specialists or other specialties if needed*. In this study, we aimed to review the clinical utility of reporting variants of uncertain significance by collecting outcomes from the reported cases. We report the number that have been upgraded or downgraded with new evidence and describe the evidence used for these changes.

## 2 Methods

### 2.1 Ethical Approval

Collection of these data has the ethical approval of the GOSH clinical audit department (ref 2781).

### 2.2 Patients

The North Thames Genomic Laboratory Hub in London performs ES to eligible cases in half of England, covering a population of over 25 million people. Eligibility for ES includes fetuses with multiple, multisystem, major structural and selected isolated abnormalities detected on fetal imaging where multidisciplinary review (involving clinical genetics, tertiary fetal medicine specialists, clinical scientists and relevant pediatric specialists) considers that a monogenic etiology is likely and molecular diagnosis may influence pregnancy or early neonatal management in the index pregnancy. Specific phenotypes eligible for ES include suspected skeletal dysplasia where placental insufficiency induced fetal growth restriction is deemed unlikely, large echogenic kidneys, major central nervous system anomalies (excluding neural tube defects), nuchal translucency greater than 6.5 mm with at least one additional anomaly and non‐immune fetal hydrops [23]. Written consent was obtained from the parents prior to testing. This consent process involves discussion regarding the possibility that the parents may receive uncertain results and/or incidental findings, with no option to opt out from these being reported.

Appendix 1 of Chandler et al. (2022) [11] describes the process that the laboratory employs for antenatal trio ES and analysis using a fetal anomaly panel. Variant classification is performed according to the ACGS guidelines (versions 2020 [24] until August 2024 and 2024 [4] following this). Warm/hot VUS (scoring between 2–5 using the 2024 guidelines but critically where only one further piece of evidence criteria would be sufficient to elevate the classification to likely pathogenic or pathogenic) that have the potential to explain the phenotype in the fetus are taken to multidisciplinary team discussions to determine whether they should be reported. When the MDT decides to report the VUS details regarding what further information may lead to a change in the VUS status are included in the report, for example any additional testing that may be useful.

All reported variants have to meet the internal quality control checks determined by internal audits to be reported without Sanger confirmation testing. If they fail to meet these criteria, variant confirmation by means of Sanger sequencing using standard protocols is undertaken prior to reporting.

The laboratory maintains a database of fetuses that have undergone ES. This database was searched from October 2020 (when the service started) to April 2025 to ascertain all cases where a VUS was reported. Outcomes of those pregnancies with a VUS were sought from their local clinical teams to determine whether the reported prenatal VUS status had been adjusted after delivery.

## 3 Results

**FIGURE 1:**
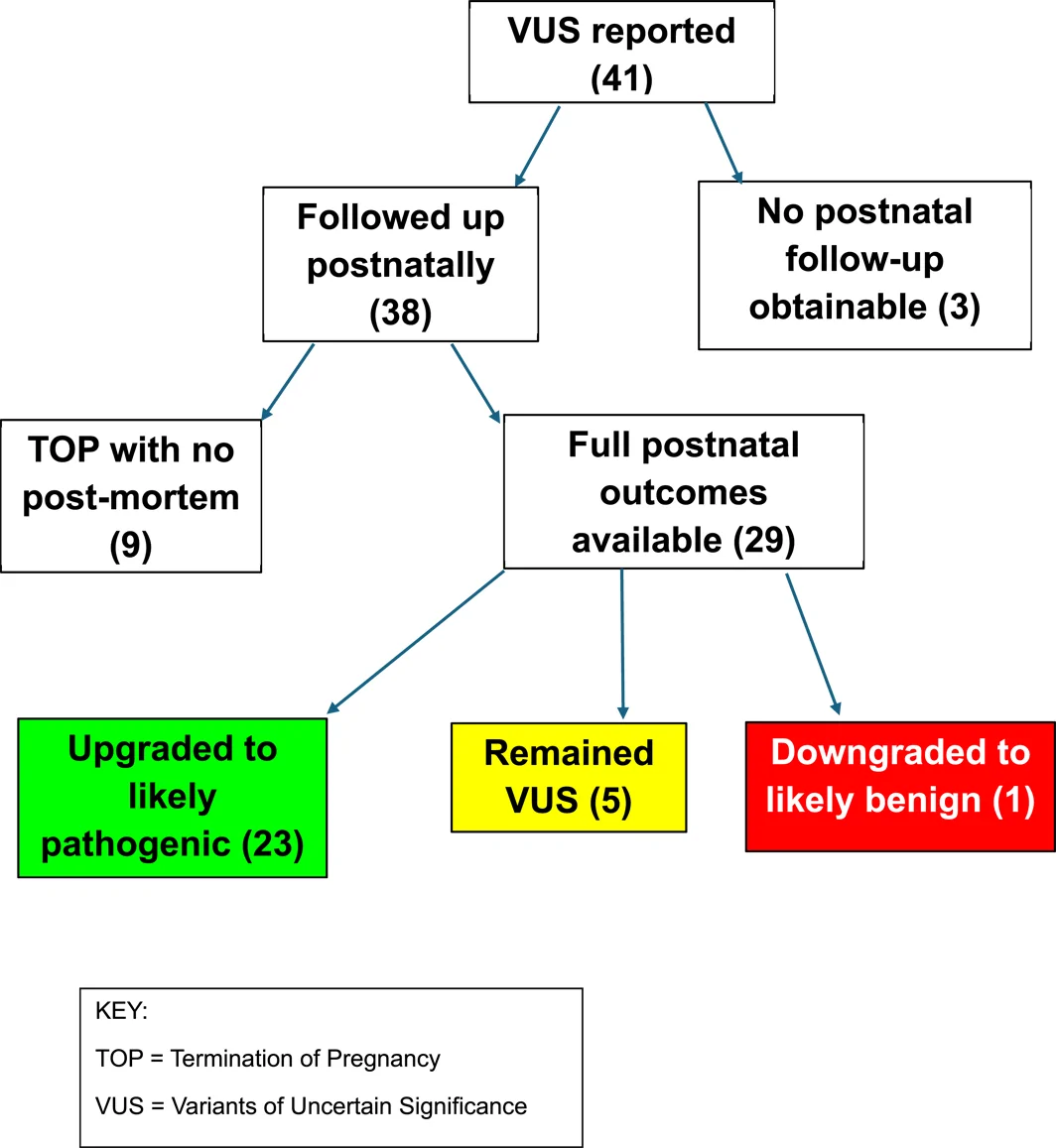
Outcomes from all the variants of uncertain significance reported.

In total, there were 881 fetal exomes sequenced between October 2020 and April 2025. Seventy (8%) cases were referred for MDT discussion and VUS were reported in forty‐one (5%) fetuses (VUS reported in 59% of cases discussed in MDT). Figure 1 shows the outcome of the cases where VUS were reported. Pre‐ or postnatal follow‐up data was available for thirty‐eight (93%) cases, of which nine (22%) were either terminated or demised in utero with postmortem declined. Of the remaining twenty‐nine cases with postnatal outcomes assessed, twenty‐three (56% of total; 79% of those with full follow‐up, including post‐mortem where relevant) were subsequently upgraded to likely pathogenic, with one (2% of total; 3% of those with follow‐up data) downgraded to likely benign (see Table 1). Five (12% of total; 17% of those with follow‐up data) cases remained classified as VUS postnatally.

**TABLE 1:** List of VUS reported where the classification was subsequently changed.

Upgraded reason	Gene and syndrome
Evolving prenatal phenotype (1)	*CCND2:* Megalencephaly‐polymicrogyria‐polydactyly‐hydrocephalus syndrome‐3 [11]
New features identified postnatally (12)	*AGPS:* Rhizomelic chondrodysplasia punctata, type 3
	*PHGDH*: Neu‐Laxova syndrome
	*DYNC2H1:* Jeune's syndrome
	*SMC3:* Cornelia de lange syndrome
	*L1CAM:* L1 syndrome
	*PIGV*: Hyperphosphatasia with mental retardation syndrome 1
	*TSC2:* Tuberous sclerosis 2
	*RASA1:* Capillary malformation‐arteriovenous malformation 1
	*ARSL:* Brachytelephalangic CDP
	*MFSD2A:* Neurodevelopmental disorder, progressive microcephaly, spasticity, and brain abnormalities
	*DYNC2H1:* Short‐rib thoracic dysplasia 3 with or without polydactyly
	*CHD7:* CHARGE syndrome
Wider family testing and protein modeling (1)	*L1CAM: L1CAM*‐related disorder
Amniotic fluid metabolic testing (1)	*GUSB:* Mucopolysaccharidosis type VII [25]
RNA studies on paternal blood (recessive condition—carrier) (1)	*PIEZO1*: Lymphatic malformation 6 (MIM 616843)
New published data (1)	*PIK3C2A:* Oculoskeletodental syndrome
New features after ToP and postmortem (4)	*SOX9:* Campomelic dysplasia
	*POMGNT1: POMGNT1*‐related disorder
	*DYNC2H1:* Short‐rib thoracic dysplasia 3 with or without polydactyly
	*ACTC1:* Dilated cardiomyopathy
Testing of donor sample to confirm *de novo &* new features identified postnatally (1)	*ZC4H2*: Wieacker‐wolff syndrome, female restricted
New features after ToP and postmortem & published data (1)	*BICD2:* Spinal Muscular atrophy [26]
**Downgraded Reason**	**Gene and syndrome**
New population data sets showed variant in healthy population (1)	*TAF1:* X‐linked syndromic intellectual developmental disorder‐33

Table 1 provides a summary of the category of evidence used for upgrade. More details on the cases along with the classification criteria applied can be found in Table S1. Of the 23 variants where a VUS was upgraded, new phenotypic features were contributory in 19 (83%). This occurred at three different time points: new imaging findings in an ongoing pregnancy (*n* = 1), postnatal phenotypic features (*n* = 13) and features found on postmortem following termination of pregnancy (*n* = 5). Follow up testing contributed to variant upgrade in four cases (17%). This included testing of a wider family (*n* = 1), obtaining a sample from an egg donor (*n* = 1), RNA testing (*n* = 1) and metabolic testing (*n* = 1). New literature or information provided by other laboratories contributed to variant upgrade in three cases (13%). For the variant that was downgraded, this was due to new data in the population database gnomAD (version 2&3 [6] vs. version 4 [27]). In three cases (13%), multiple pieces of evidence were used to upgrade the variant.

For those 23 cases where the initial VUS reported was subsequently upgraded, median time from initial VUS report to variant upgrade was 226 days, mean time was 344 days. Minimum time period was 2 days and the maximum time period was 1696 days.

## 4 Discussion

Our data supports the reporting of selected VUS identified prenatally after an MDT review. Out of the 27 VUS reported by the North Thames Genomic Laboratory Hub with full postnatal follow‐up data, 23 were upgraded postnatally. Even when including those cases that were either lost to follow up or declined postmortem, over half of all VUS reported were subsequently upgraded. A similar study reporting VUS outcomes from a set of Canadian commercial laboratories performed in 2022 found a much lower yield for subsequent upgrading [10]. That investigation identified that out of 93 cases undergoing ES, 39 (42%) had at least one VUS reported (with one of the commercial laboratories included reporting VUS in 70% of their cases). This was much higher than our laboratory where only 5% of cases had a VUS reported. Of the Canadian study's 39 cases with a VUS reported, only 13% (5/39) were subsequently deemed to be significant after postnatal follow‐up. This difference is likely to be due to a lower threshold for reporting VUS and the fact that testing was done in commercial laboratories with no prior MDT discussion.

Chromosomal microarray is another form of prenatal genetic testing for fetuses with ultrasound abnormalities and its use has been established for over a decade [28]. VUS can also be reported prenatally with this investigation. Awareness of the impact to parents of VUS reporting with well‐established microarray testing is relevant when considering the value of VUS reporting in more novel prenatal sequencing. McCoy et al. [29] followed up families that received a prenatal VUS report on microarray and found that compared to a control group of parents who did not receive prenatal genetic testing, parents who had received a prenatal VUS diagnosis were no more likely to have concerns about their child's development by the time that child was around 6 years old. In this cohort, only 8.5% of VUS reported prenatally were subsequently reclassified as pathogenic, yet parents were not found to be overanxious about their children in their early years. Shi et al. [30] found that parents receiving a VUS reported from a microarray that was de novo were significantly more likely to terminate the pregnancy (58% of cases (18/31)) compared to VUS reported that were inherited from at least one parent (5% of cases (5/108)). This underlines the nuances of receiving a VUS report and the importance of adequate counseling and follow up to ensure that parents are enabled to make the most appropriate decisions for themselves and their families.

There are multiple studies that emphasise the importance of an MDT review prior to the reporting of VUS [10, 31, 32]. Micke et al. [32] in 2022 described an extensive MDT including “fetal medicine specialists, medical geneticists, genetic counsellors, neonatologists, bioethicists, and translational researchers” to reclassify half of the VUS that were to be reported initially. The MDT employed by our laboratory is similar in composition to that described by the MDT assembled in the Netherlands by Diderich et al. 2023 (“clinical geneticists and laboratory specialists in clinical genetics… Fetal medicine specialists are not routinely present… however they are consulted … per particular cases and if necessary these cases are discussed in another regular weekly meeting of fetal medicine specialists where clinical geneticists are also present”) [31]. They found that this approach resulted in the reporting of VUS in 2% of cases, comparable to our VUS reporting rate of 5%. Their study reported nine VUS, with three (33%) upgraded to significant and two (22%) downgraded to benign.

One major concern for reporting VUS antenatally is that it may lead to the parents deciding to terminate a pregnancy that in reality carries a benign variant (although it should be noted that parents will not often decide to terminate solely on the basis of a genetic abnormality, but also on the basis of the fetal ultrasound findings and many other factors associated with their personal backgrounds and circumstances). We found that one case reported as VUS from our laboratory was subsequently downgraded after delivery (see Table 1). This was a case referred at 25 weeks gestation after fetal ultrasound scan demonstrated severe intrauterine growth restriction with no evidence of placental compromise. The male fetus was found to be hemizygous for a *TAF1* c.92 A > T p.(Gln31Leu) variant that had been maternally inherited (mother was heterozygous). Hemizygous pathogenic variants in *TAF1* cause “X‐linked syndromic intellectual developmental disorder‐33” which is associated with fetal growth restriction [32]. The variant was not present in the mother's unaffected father and brother. At the time that this ES report was generated, the identified variant was present in 1 and 3 heterozygous females in the gnomad v2.1 and v3.1.2 databases, respectively and not seen in any males (PM2_moderate). The missense constraint score was 5.49 (PP2_supporting); however, in silico tools predicted that the variant should be tolerated (BP4_supporting). It was therefore reported to the parents as a VUS. After birth, it was noted that the baby did not exhibit clinical features consistent with a pathological *TAF1* variant, such as dysmorphic facies. A repeat review using the updated gnomADv4.1.0 database, almost 1 year after the initial report was generated, showed the variant to be present in 27 assumed unaffected individuals, resulting in a reclassification of the VUS to likely benign (BS2_strong; BP4_supporting). As population databases increase in size, the frequency of inappropriate identification of VUS should decrease [33]. The prenatal phenotype of severe growth restriction was very non‐specific which also hindered the process of variant interpretation.

It has often been argued that prenatal ES poses additional challenges compared with postnatal assessment because of a potentially limited phenotype detectable in utero. Although prenatal ES in fetuses with multiple abnormalities has been demonstrated to be effective in identifying pathological variants [34], there remains a role for autopsy in those cases that result in termination, stillbirth, or neonatal death to refine the phenotype. Godbole et al. [35] found that autopsy of fetuses that were either terminated or died in the second trimester of pregnancy provided additional information in over 60% of cases, with just under 25% of autopsies providing findings required for a final diagnosis. Our data support this finding as five fetuses with a prenatally reported VUS had the classification upgraded after termination and autopsy identified additional features. Variants identified in further nine fetuses who underwent termination but did not have an autopsy were classified as VUS. If the parents decline an autopsy, other postmortem investigative options which may be more acceptable [36, 37] such as physical examination, photography or minimally/non‐invasive autopsy [38, 39] may provide the additional information required to reclassify a VUS.

Whilst 18/23 (78%) of variants were upgraded with evidence that included postnatal phenotypic information, there were a number of other strategies employed to upgrade variants with multiple strategies being utilised for 3/23 (13%) variants (see Table 1, *BICD2*, *ZC4H2 & L1CAM*). In one case, the evolving prenatal phenotype on follow‐up scans provided enough evidence to upgrade the variant (Table 1, *CCND2*) because the neurological features of the condition had not developed at the gestation of testing [40]. This then informed pregnancy management as the patient had a TOP following the diagnosis. This shows the importance of follow‐up scans and not just waiting for post‐natal phenotypic information. New published data contributed in two cases highlighting the importance of publishing novel prenatal phenotype‐genotype or mechanisms of disease and the impact this is having on eliminating the uncertainty for other parents. Submission of variants to databases such as ClinVar [7] is also a useful aid to variant classification. Additional testing and protein modeling was also utilised in 4/23 (17%), as technology develops with the movement toward omics technologies, it is likely that this area will rapidly develop. Studies on the utility of these technologies in the prenatal setting are required.

There are some limitations to this study. Although we know that over 50% of all the reported VUS cases were subsequently upgraded, we do not know postnatal outcomes from 12 (29%) of our included cases (nine had a termination with no postmortem and three were lost to follow up). We found only one example of a VUS being downgraded from those cases for which we had postnatal follow up.

## 5 Conclusion

Here we demonstrate the clinical utility of reporting VUS from prenatal sequencing following multidisciplinary team discussion as this alerts the healthcare teams of the need to be vigilant and follow‐up cases both pre‐ and postnatally to seek findings that can alter variant classification. Although VUS leave parents with uncertainty, we found that follow up can resolve this in > 50% of cases, providing a diagnosis and access to informed counseling, improved management and determination of recurrence risk for future pregnancies.

In summary, our data show that if MDT discussion, that includes prenatal geneticists, clinical scientists and fetal medicine experts for phenotyping, agrees that the VUS is a phenotypic fit and minimal additional information is required for upgrade, follow‐up data, be it from postmortem, postnatal examination, or close follow‐up in pregnancy, can resolve the status in > 50% of cases. In these circumstances, the follow‐up or additional testing suggested should be included in the report.

## Funding

The authors have nothing to report.

## Conflicts of Interest

The authors declare no conflicts of interest.

## Supporting information


**Table S1:** Detailed information on the VUS cases that were subsequently reclassified with reasoning for reclassification.

## Data Availability

The data that support the findings of this study are available from the corresponding author upon reasonable request.
