# Supplementary material for: Is There Potential Clinical Utility in Reporting Variants of Uncertain Significance From Prenatal Sequencing?
Source: Prenat Diagn. 2026 Jun 18;46(10):1557–64. doi: 10.1002/pd.70192 (PMC13571796; doi:10.1002/pd.70192)
Supplement: Supplementary file 1 [file PD-46-1557-s001.docx]

| Case | Consanguinity | GA | Prenatal phenotype (HPO terms) | Variant | Inheritance | Criteria applied for initial VUS reported variant | Phenotype (OMIM) | Pregnancy Outcome | Reason for reclassification | Updated phenotype (HPO term) | Arguments for reclassification of the variant | Criteria applied post reclassification |
| --- | --- | --- | --- | --- | --- | --- | --- | --- | --- | --- | --- | --- |
| 1 | No | 20 weeks | Postaxial hand polydactyly (0001162); Postaxial foot polydactyly (0001830); Partial agenesis of the corpus callosum (0001338); Hyperechogenic kidneys (0004719); Enlarged kidney (0000105); Ventricular septal defect (0001629) | *CCND2* NM_001759.4:c.860_861delinsAA, p.(Ile287Lys) het | AD De novo | PM2_moderate PP3_supporting PS2_moderate  (VUS) | Megalencephaly-polymicrogyria-polydactyly-hydrocephalus syndrome 3 (MIM 615938) | TOP at 31 weeks | Prenatal update of phenotype at ultrasound scan at 28 weeks' gestation | Macrocephaly (0000256); Polymicrogyria (0003577) | Updated phenotype at 28 weeks enabled application of PP4_supporting and upgrading to Class 4 Likely Pathogenic | PM2_moderate PP3_supporting PS2_moderate  PP4_supporting  (Likely pathogenic) |
| 2 | No | 21 weeks | Fetal skin edema (0025672); Fetal pleural effusion (0025676); Fetal ascites (0001791) | *BICD2*  NM_001003800.2:c.1624T>G p.(Cys542Gly) het | AD De novo | PM2_moderate PP3_supporting PM5_supporting  PS2_supporting  (VUS) | Spinal muscular atrophy, lower extremity-predominant, 2A (MIM 615290) and 2B (MIM 618291) | TOP before 24 weeks | Another publication reporting hydrops with BICD2 related disease (33686258) Update of phenotype after postmortem confirmed clinical features consistent with those described in severe *BICD2* variants | Arthrogryposis multiplex congenita (0002804); Myopathy (0003198) | Another publication reporting hydrops with BICD2 related disease & Updated phenotype after postmortem - upgrade PS2_moderate (PMID: 35506549)^22^ | PM2_moderate PP3_supporting PM5_supporting  PS2_moderate  (Likely pathogenic) |
| 3 | Yes (third cousins) | 18 weeks | Fetal pleural effusion (0025676); Fetal ascites (0001791); Hyperechogenic kidneys (0004719); Increased nuchal translucency (0010880); Short femur (0003097), Flat facial profile (0012368); Abnormal coccyx morphology (0008519) | *PIK3C2A* NM_002645.3:c.2684dup p.(Leu895Phefs) hom | AR parents both het | PVS1_moderate (limited evidence for LOF as mechanism for disease – three reported variants in consanguineous families) PM2_moderate  (VUS) | Oculoskeletodental syndrome (MIM 618440) | Livebirth, emergency Caesarean Section at 31+6 weeks for premature rupture of membranes and placental abruption | Postnatal update with new phenotypical developments within the first three years of life and new paper (PMID: 35770347) with more cases with LOF variants. | Megalocornea (0000485); Atresia of the external auditory canal (0000413); Micrognathia (0000347); Bicornuate uterus (0000813); Abnormal facial shape (0001999); Global developmental delay (0001263); Dysphagia (0002015); Short stature (0004322); Unilateral renal agenesis (0000122); Hydronephrosis (0000126); Skeletal dysplasia (0002652) | Updated postnatal phenotype enabled application of PVS1 _strong, PM3_supporting and PP4_supporting, upgrading to Class 4 Likely Pathogenic. | PVS1 _strong, PM2_moderate PM3_supporting PP4_supporting  (Likely pathogenic) |
| 4 | No | 28 weeks | Short long bone (0003026); Bowed humerus (0003865); Decreased head circumference (0040195); Single umbilical artery (0001195) | *AGPS* NM_003659.4:c.1255C>T p.(Arg419Cys) hom | AR parents both het | PP3_supporting PP2_supporting PM2_moderate PM5_Supporting  (VUS) | Rhizomelic chondrodysplasia punctata, type 3 (MIM 600121) | Livebirth, at term | Postnatal update with new phenotypical developments and metabolic investigation results | Rhizomelic arm shortening (0004991); Rhizomelic leg shortening (0012106); Coronal cleft vertebrae (0003417); Decreased circulating plasmalogen concentration (4000165) | Updated postnatal phenotype enabled application of PP4_strong, upgrading to Class 4 Likely Pathogenic. | PP3_supporting PP2_supporting PM2_moderate PM5_Supporting  PP4_strong  (Likely pathogenic) |
| 5 | No | 20 weeks | Hand clenching (0001188); Cataract (0000518); Micrognathia (0000347); Agenesis of corpus callosum (0001274); Scoliosis (0002650);  Hemivertebrae (0002937); Absence of the sacrum (0010305); Rocker bottom foot (0001838); Microcephaly (0000252); Pontocerebellar atrophy (0006879) | *PHGDH* NM_006623.4:c.56G>T p.(Cys19Phe) het & NM_006623.4:c.1572_1575dup p.(Thr526Cysfs*3) het | AR confirmed compound het | c.56G>T p.(Cys19Phe):  PM2_moderate PP3_supporting (VUS)  c.1572_1575dup p.(Thr526Cysfs*3): PM2_moderate PVS1_moderate (frameshift is in last exon removing 0.9% of protein)  (VUS) | Neu-Laxova syndrome (MIM 256520) | TOP at 26 weeks | Update of phenotype after postmortem confirmed clinical features consistent with Neu-Laxova syndrome | Hypertelorism (0000316); Low-set ears (0000369); Narrow mouth (0000160); Short neck (0000470); Hand clenching (0001188); Syndactyly (0001159); Multiple joint contractures (0002828); Focal polymicrogyria (0032471); Abnormal pons morphology (0007361); Cerebellar hypoplasia (0001321) | Updated phenotype after post mortem enabled application of PP4_moderate for both variants and PM3_moderate for the c.56G>T p.(Cys19Phe) variant, enabling upgrading to Class 4 Likely Pathogenic | c.56G>T p.(Cys19Phe):  PM2_moderate PP3_supporting  PM3_moderate  PP4_moderate (Likely pathogenic)  c.1572_1575dup p.(Thr526Cysfs*3) PM2_moderate PVS1_moderate  PP4_moderate  (Likely pathogenic) |
| 6 | No | 26 weeks | Flat facial profile (0012368) | *ARSL* NM_000047.3:c.1162C>T p.(Arg388Cys) hemi | XL, mother het | PP3-supporting PM2-moderate PP4_supporting  (VUS) | Chondrodysplasia punctata (MIM: 302950) | Livebirth, at term | Postnatal update with new phenotypical developments | Stippling of the epiphyses of the distal phalanges of the hand (0010255) | Updated postnatal phenotype enabled application of PP4_strong, upgrading to Class 4 Likely Pathogenic. | PP3-supporting PM2-moderate PP4_strong  (Likely pathogenic) |
| 7 | Yes (second cousins) | 22 weeks | Short long bone (0003026); Rhizomelic arm shortening (0004991); Rhizomelic leg shortening (0012106) | *DYNC2H1* NM_001080463.2:c.3847G>C p.(Asp1283His) Hom | AR parents both het | PM2_moderate PP3_supporting PM3_supporting PP4_supporting  (VUS) | Short-rib thoracic dysplasia 3 with or without polydactyly (MIM 613091) | Livebirth, emergency Caesarean Section at term | Postnatal update with new phenotypical developments | Cone-shaped epiphysis (0006059); Horizontal ribs (0000888) | Updated postnatal phenotype enabled application of PM3_moderate and PP4_moderate, upgrading to Class 4 Likely Pathogenic. | PM2_moderate PP3_supporting PM3_supporting PP4_moderate  (Likely pathogenic) |
| 8 | No | 20 weeks | Thickened nuchal skin fold (0000474); Prenasal oedema (0000282);Micrognathia (0000347); Deep philtrum (0002002) | *SMC3* NM_005445.3:c.1162_1164del p.(Lys388del) het | AD De novo | PM4_supporting PM2_moderate  PS2_supporting  (VUS) | Cornelia de Lange syndrome 3 (MIM: 610759) | Livebirth, at term | Postnatal update with new phenotypical developments | Short stature (0004322); Microcephaly (0000252); Hypotonia (0001252); Synophrys (0000664); Hypertrichosis (0000998); Posteriorly rotated ears (0000358); Low-set ears (0000369) | Updated postnatal phenotype enabled application of PS2_moderate and PP4_supporting, upgrading to Class 4 Likely Pathogenic. | PM4_supporting PM2_moderate  PS2_moderate  PP4_supporting  (Likely pathogenic) |
| 9 | No | 21 weeks | Ventriculomegaly (0002119); Micrognathia (0000347); Cerebellar vermis hypoplasia (0001320); Partial agenesis of the corpus callosum (0001338); Absent septum pellucidum (0001331) | *L1CAM* NM_001278116.2:c.400G>A p.(Gly134Ser) hemi | XL mother het | PM2_moderate PP3_supporting  (VUS) | Partial agenesis of corpus callosum (MIM 304100), CRASH syndrome (MIM 303350), syndromic hydrocephalus (MIM 307000) and MASA syndrome (MIM 303350) | Livebirth, at term | Postnatal update with new phenotypical developments | Adducted thumb (0001181); Macrocephaly (0000256); Hypoplasia of the pons (0012110) | Updated postnatal phenotype enabled application of PP4_strong, upgrading to Class 4 Likely Pathogenic. | PM2_moderate PP3_supporting  PP4_strong  (Likely pathogenic) |
| 10 | Yes (first cousins) | 19 weeks | Thickened nuchal skin fold (0000474); Hyperechogenic kidneys (0004719); Enlarged kidney (0000105); Small face (0000274) | *PIGV*  NM_017837.4:c.905T>C p.(Leu302Pro) hom | AR (parents both het) | PM2_moderate PP3_supporting PM3_supporting PP4_supporting  (VUS) | Hyperphosphatasia with mental retardation syndrome 1 (MIM 239300) | Born at term. Deceased on day of delivery due to pulmonary hypoplasia | Postnatal update with new phenotypical developments | Polycystic kidney dysplasia (0000113); Renal dysplasia (0000110); Pulmonary hypoplasia (0002089); Anal atresia (0002023); Cleft palate (0000175); Elevated circulating alkaline phosphatase concentration (0003155); Aplasia/Hypoplasia of the distal phalanges of the hand (0009835); Talipes (0001883); Ventricular septal defect (0001629); Left ventricular outflow tract obstruction (0032092); Wide nasal bridge (0000431); Broad nasal tip (0000455); Tented upper lip vermilion (0010804); Hypertelorism (0000316) | Updated postnatal phenotype enabled application of PM3_moderate and PP4_moderate, upgrading to Class 4 Likely Pathogenic. | PM2_moderate PP3_supporting PM3_**moderate** PP4_moderate  (Likely pathogenic) |
| 11 | No | 20 weeks | Fixed elbow flexion (0006471); Wrist flexion contracture (0001239); Knee extension contracture (0034672); Talipes (0001883); Overlapping fingers (0010557) | *ZC4H2* NM_018684.3:c.607T>C p.(Cys203Arg) het | AD – duo (egg donor) not in father | PM2_moderate PP3_supporting  (VUS) | Wieacker-Wolff syndrome, female restricted (MIM 301041) | TOP after 24 weeks | Testing of egg donor to confirm variant had arisen de novo. Update of phenotype after post-mortem. | Arthrogryposis multiplex congenita (0002804) | Updated postnatal phenotype enabled application of PS2-moderate and PP4_supporting, upgrading to Class 4 Likely Pathogenic. | PM2_moderate PP3_supporting  PS2-moderate  PP4_supporting  (Likely pathogenic) |
| 12 | No | 12 weeks | Increased nuchal translucency (0010880); Short femur (0003097); Short humerus (0005792); Absent in utero ossification of vertebral bodies (0008435) | *SOX9* NM_000346.4:*c*.356C>A p.(Ala119Glu) het | AD – mother mosaic | PP3-supporting  PM2-moderate PM5-supporting PS2-supporting  (VUS) | Campomelic dysplasia (MIM 114290) | TOP before 24 weeks | Update of phenotype after post-mortem | Tibial bowing (0002982); Femoral bowing (0002980); Short 1st metacarpal (0010034); Hypoplastic scapulae (0000882); Talipes equinovarus (0001762) | Updated postnatal phenotype enabled application of PP4-moderate, upgrading to Class 4 Likely Pathogenic. | PP3-supporting  PM2-moderate PM5-supporting PS2-supporting  PP4-moderate  (Likely pathogenic) |
| 13 | No | 24 weeks | Fetal skin edema (0025672); Fetal pleural effusion (0025676); Fetal ascites (0001791) | *GUSB* NM_000181.4:c.1270C>T p.(His424Tyr) het  & NM_000181.4:c.953A>G p.(Asp318Gly) het | AR. Egg donor.  Confirmed *in trans* by long-read sequencing | *GUSB* c.1270C>T:  BP4-supporting PM2-moderate  PM3-Strong (Likely pathogenic)  *GUSB* c.953A>G PP3-supporting PM2-moderate PM3-moderate  (VUS) | Mucopolysaccharidosis VII (MIM 253220) | Livebirth at 36+5 weeks by emergency Caesarean Section | Biochemical testing of amniotic fluid glycosaminoglycans confirmed presence of dermatan sulphate, in keeping with diagnosis of Mucopolysaccharidosis VII (MIM 253220). (PMID: 37964423) | Abnormality of glycosaminoglycan metabolism (0004371) | Biochemical testing findings allowed application of PP4-strong, upgrading to Class 4 Likely Pathogenic. | *GUSB* c.1270C>T:  BP4-supporting PM2-moderate  PM3-Strong  PP4_strong (Pathogenic)  *GUSB* c.953A>G PP3-supporting PM2-moderate PM3-moderate  PP4_strong  (Likely pathogenic) |
| 14 | No | 24 weeks | Cleft lip (0410030); Cleft palate (0000175); Abnormal ventricular septum morphology (0010438) | *TSC2* NM_000548.5:c.910T>C p.(Trp304Arg) het | AD – maternally inherited | PM2_Moderate PP3_Supporting  PS4_Moderate  (VUS) | Tuberous Sclerosis 2 (MIM 613254) | Livebirth at 35 weeks due to maternal pre-eclampsia | Postnatal update with new phenotypical developments. Mother also found to have clinical features of Tuberous sclerosis 2 (renal cyst and MRI head changes) | Cardiac rhabdomyoma (0009729) | Updated postnatal phenotype alongside confirmation of diagnosis in mother enabled application of PP4-Supporting, upgrading to Class 4 Likely Pathogenic. | PM2_Moderate PP3_Supporting  PS4_Moderate  PP4_supporting  (Likely pathogenic) |
| 15 | No | 32 weeks | Fetal pleural effusion (0025676); Fetal ascites (0001791); Polyhydramnios (0001561) | *RASA1* NM_002890.3:c.1193G>A p.(Arg398Gln) het | AD – paternally inherited | PM2_Moderate PP2_Supporting PP3_Supporting  (VUS) | Capillary malformation-arteriovenous malformation 1 (MIM 608354) | Livebirth at 35 weeks | Postnatal update with new phenotypical developments. Father declined examination. | Chylothorax (0010310); Capillary malformation (0025104); Hemihypertrophy of lower limb (0100553) | Updated postnatal phenotype enabled application of PP4-moderate, upgrading to Class 4 Likely Pathogenic. | PM2_Moderate PP2_Supporting PP3_Supporting  PP4_moderate  (Likely pathogenic) |
| 16 | No | 22 weeks | Partial agenesis of the corpus callosum (0001338); Inferior cerebellar vermis hypoplasia (0007068) | *L1CAM* NM_001278116.1:c.2168G>A p.(Gly723Glu) hemi | XL mother het | PM2_moderate (VUS) | Partial agenesis of corpus callosum (MIM 304100), CRASH syndrome (MIM 303350), syndromic hydrocephalus (MIM 307000) and MASA syndrome (MIM 303350) | TOP before 24 weeks | Review of predicted structural impact of the *L1CAM* variant and testing of maternal grandparents showed variant was *de novo* in the mother. | Nil | Review of predicted structural impact of the *L1CAM* variant enabled application of PM1-moderate, upgrading to Class 4 Likely Pathogenic. | PM2_Moderate  PS2_Moderate  PM1_Moderate  (Likely pathogenic) |
| 17 | No | 21 weeks | Fetal skin edema (0025672); Fetal pleural effusion (0025676); Fetal ascites (0001791) | *PIEZO1* NM*_*001142864.4:c.5801+5_5801+8del het  & NM*_*001142864.4:c.5907_5909del p.(Val1970del) het | AR confirmed compound het | c.5801+5_5801+8del:  PM2 moderate  PP3 supporting PP4 supporting  (VUS)  c.5907_5909del: PM2 moderate PM4_supporting PP4 supporting  (VUS) | Lymphatic malformation 6 (MIM 616843) | Stillbirth | mRNA splice site analysis of paternal variant predicted the transcript to undergo nonsense mediated decay | Nil (postmortem not carried out) | Information after mRNA splice site analysis allowed the paternal variant to be upgraded to pathogenic with PVS1_very strong and PM3_moderate applied. The maternally inherited variant was upgraded to likely pathogenic with the additional application of PM3_moderate. | c.5801+5_5801+8del:  PM2 moderate  PP4 supporting PVS1_very strong  PM3_moderate  (Pathogenic)  c.5907_5909del:  PM2 moderate PM4_supporting PP4 supporting  PM3_moderate  (Likely pathogenic) |
| 18 | No | 21 weeks | Agenesis of corpus callosum (0001274); Mild fetal ventriculomegaly (0010952); Cerebellar hypoplasia (0001321); Microcephaly (0000252) | *MFSD2A* NM_032793.5:c.253_255del p.(Ile85del) hom | AR parents both het | PM4_supporting PM2_moderate PM3_supporting PP4_supporting  (VUS) | Neurodevelopmental disorder with progressive microcephaly, spasticity, and brain abnormalities (MIM 616486) | Livebirth at term | Postnatal update with new phenotypical developments | Progressive macrocephaly (0004481); Aqueductal stenosis (0002410); Cerebral atrophy (0002059); Dystonia (0001332); Infantile spasms (0012469) | Updated postnatal phenotype enabled application of PP4-moderate, upgrading to Class 4 Likely Pathogenic. | PM4_supporting PM2_moderate PM3_supporting PP4_moderate  (Likely pathogenic) |
| 19 | No | 36 weeks | Short long bone (0003026); Narrow chest (0000774) | *DYNC2H1* NM_001080463.2:c.4127+2T>G het  & NM_001080463.2:c.7220C>T p.(Ser2407Leu) het | AR confirmed compound het | *DYNC2H1* c.4127+2T>G PVS1_very strong  PM2_moderate PP4_supporting  (Pathogenic)  *DYNC2H1* c.7220C>T  PM2_moderate PM3_moderate BP4_supporting PP4_supporting  (VUS) | Short-rib thoracic dysplasia 3 with or without polydactyly (MIM 613091) | Livebirth at term | Postnatal update with new phenotypical developments | Polydactyly (0010442); Short ribs (0000773); Trident acetabulum (0034374) | Updated postnatal phenotype enabled application of PP4-moderate to the *DYNC2H1* c.7220C>T p.(Ser2407Leu) variant, upgrading it to Class 4 Likely Pathogenic. | *DYNC2H1* c.4127+2T>G PVS1_very strong  PM2_moderate PP4_moderate  (Pathogenic)  *DYNC2H1* c.7220C>T  PM2_moderate PM3_moderate BP4_supporting PP4_moderate  (Likely pathogenic) |
| 20 | Yes | 33 weeks | Cerebellar vermis hypoplasia (0001320); Mild fetal ventriculomegaly (0010952); Hypoplasia of the pons (0012110); Abnormal midbrain morphology (0002418) | *POMGNT1* NM_017739.4:c.1396T>C p.(Trp466Arg) hom | AR parents both het | PM2_moderate PP3_supporting PM3_supporting  (VUS) | Muscular dystrophy-dystroglycanopathy (congenital with brain and eye anomalies), type A, 3 (MIM 253280) | Livebirth at term | Postnatal update with new phenotypical developments, information from other testing laboratories in England & use of gnomADv2.1 regional missense constraint score | Cortical dysplasia (0002539); Parasagittal parieto-occipital polymicrogyria (0032415), Retinal detachment (0000541); Cerebellar cyst (0002350); Ophthalmoplegia (0000602) | Updated postnatal phenotype enabled application of PM3_moderate and PP2_supporting, upgrading to Class 4 Likely Pathogenic. | PM2_moderate PP3_supporting PM3_moderate  PP2_supporting  (Likely pathogenic) |
| 21 | No | 25 weeks | Thickened nuchal skin fold (0000474); Facial edema (0000282); Fetal ascites (0001791); Congenital pulmonary airway malformation (0010959) | *CHD7* NM_017780.4:c.4034G>A p.(Arg1345His) het | AD – paternally inherited | PM2_moderate PS4_supporting PP3_supporting PP2_supporting  (VUS) | CHARGE syndrome (MIM 214800) | Livebirth at term | Postnatal update with new phenotypical developments | Micropenis (0000054); Postnatal growth retardation (0008897) | Updated postnatal phenotype enabled application of PP4-moderate, upgrading to Class 4 Likely Pathogenic. | PM2_moderate PS4_supporting PP3_supporting PP2_supporting  PP4_moderate  (Likely pathogenic) |
| 22 | No | 21 weeks | Micromelia (0002983); Tibial bowing (0002982) | *DYNC2H1* NM_001080463.2:c.6614G>A p.(Arg2205His) het  & NM_001080463.2:c.11483T>C p.(Leu3828Ser) het | AR confirmed compound het | c.6614G>A PM3_strong  PM2_moderate  PP2_supporting  PP4_supporting  (likely pathogenic)  c.11483T>C PM2_moderate  PM3_moderate  PP4_supporting  (VUS) | Short-rib thoracic dysplasia 3 with or without polydactyly (MIM 613091) | TOP after 24 weeks | Update of phenotype after post-mortem | Short ribs (0000773) | Updated postnatal phenotype enabled application of PP4-moderate, upgrading to Class 4 Likely Pathogenic. | c.6614G>A PM3_strong  PM2_moderate  PP2_supporting  PP4_moderate  (likely pathogenic)  c.11483T>C PM2_moderate  PM3_moderate  PP4_moderate  (Likely pathogenic) |
| 23 | No | 20 weeks | Increased nuchal translucency (0010880); Right ventricular hypertrophy (0001667); Talipes (0001883) | *ACTC1* NM_005159.4:c.595G>A p.(Gly199Ser) het | AD De novo | PM2_moderate  PP3_supporting  PP2_supporting  PS2_supporting  (VUS) | Dilated cardiomyopathy (MIM: 613424, 612098) | TOP | Update of phenotype after post-mortem | Abnormal cardiomyocyte morphology (0031331) | Updated postnatal phenotype enabled application of PS2-moderate, upgrading to Class 4 Likely Pathogenic. | PM2_moderate  PP3_supporting  PP2_supporting  PS2_moderate  (Likely pathogenic) |
| 24 | No | 27 weeks | Large placenta (0006267); Intrauterine growth retardation (0001511);  Abnormal umbilical cord blood vessel morphology (0011403);  Hepatic calcification (0006559) | *TAF1* NM_004606.5:c.92A>T p.(Gln31Leu) hemi | XL mother het | PM2_moderate  BP4_supporting  PP2_supporting  (VUS) | Intellectual developmental disorder, X-linked syndromic 33 (MIM 300966) | TOP | Postnatal features not in keeping with disorder & review of newer version of gnomAD (v4.1.0) showing variant hemizygous in 27 unaffected makes | N/A |  | BP4_supporting  BS2_strong  (Likely benign) |

Key – AD=autosomal dominant, AR=autosomal recessive, GA= gestation age, hemi=hemizygous, het=heterozygous, hom=homozygous, TOP=termination of pregnancy, VUS=variant of uncertain clinical significance, XL=X-linked.
